# ABA and Pre-Harvest Sprouting Differences in Knockout Lines of OsPHS3 Encoding Carotenoid Isomerase via CRISPR/Cas9 in Rice

**DOI:** 10.3390/plants14030345

**Published:** 2025-01-23

**Authors:** Yu-Jin Jung, Jiyun Go, Jin-Young Kim, Hyo-Ju Lee, Jong-Hee Kim, Hye-Mi Lee, Yong-Gu Cho, Kwon-Kyoo Kang

**Affiliations:** 1 Division of Horticultural Biotechnology, Hankyong National University, Anseong 17579, Republic of Korea; yuyu1216@hknu.ac.kr (Y.-J.J.); zino@hknu.ac.kr (J.-Y.K.); ju950114@naver.com (H.-J.L.); jonghee014@hknu.ac.kr (J.-H.K.); abc_772@naver.com (H.-M.L.); 2 Institute of Genetic Engineering, Hankyong National University, Anseong 17579, Republic of Korea; 3 Department of Bio-Environmental Chemistry, College of Agriculture and Life Sciences, Chungnam National University, Daejeon 34134, Republic of Korea; jy.go2369@gmail.com; 4 Department of Crop Science, College of Agriculture and Life & Environment Sciences, Chungbuk National University, Cheongju 28644, Republic of Korea; ygcho@chungbuk.ac.kr

**Keywords:** pre-harvest sprouting, CRISPR/Cas9, number of tillers, ABA, carotenoid biosynthesis, gene expression

## Abstract

We generated and characterized knockout mutant lines of the *OsPHS3* gene using the CRISPR/Cas9 system. The knockout lines of the *OsPHS3* gene showed that 1 bp and 7 bp deletion, early termination codons were used for protein production. Agronomic characteristics of knock-out lines were reduced in plant height, culm diameter, panicle length, seed size and weight, except for the number of tillers. In addition, we analyzed the expression levels of carotenoid biosynthesis genes by qRT-PCR. Among the genes encoding carotenoid metabolic pathway enzymes, the level of transcripts of *PSY1*, *PSY2*, *PSY3*, *PDS* and *ZDS* were higher in the KO lines than in the WT line. In contrast, transcription of the ε*-LCY*, *β*-*LCY* and *ZEP1* genes were downregulated in the KO lines compared to the WT line. Also, the KO lines decreased carotenoid content and ABA amount compared to WT, while preharvest sprouts increased. These results suggested that they would certainly help explain the molecular mechanisms of PHS in other crops, such as wheat and barley, which are susceptible to PHS.

## 1. Introduction

The most influential factors in rice production are abiotic stresses such as temperature, moisture, and light. Among them, frequent rainfall during the rice harvest season causes pre-harvest sprouting (PHS) phenomena, not only causes reduction in grains yield but also affects the quality of the grain [1,2]. As a mechanism to prevent PHS, seed dormancy is a property that limits seed germination under unfit ecological conditions [3,4]. Previous studies have shown that abscisic acid (ABA) biosynthesis or impaired-reactive mutations (corn and rice *viviparous* (*vp*)) and *Arabidopsis* ABA deficiency (*aba*), and ABA insensitivity (*abi*) are known to germinate rapidly under humid conditions [5,6]. At least 10 *viviparous* mutations have been reported in corn (*Zea may* L.), most of which (*vp2*, *vp5*, *vp7*, *vp9*, *w3*, *y9*) have blocked the biosynthesis of carotenoid precursors, resulting in poor ABA synthesis [7]. In carotenoid biosynthesis, the phytoene synthetase (PSY) enzyme produces C_40_ carotenoid phytoene in the C_20_ geranylgeranyl diphosphate (GGPP) molecule. Subsequently, the phytoene undergoes four unsaturated reactions with the production of lycopene, followed by a series of steps including cyclization and hydroxylation to produce *α*-carotene, *β*-carotene, lutein, and xanthophyll and zeaxanthin [8]. After the cleavage of the C_40_ carotenoid precursor, the intermediate xanthoxin is converted to ABA via ABA aldehyde [9,10]. 15-*cis*-phytoene obtained by phytoene synthase (PSY) is converted to all-trans lycopene through several steps of unsaturation and isomerization through the action of phytoene desaturase (PDS) and *ζ* desaturase (ZDS), 15-*cis*-ζ isomerase (Z-ISO), and carotenoid isomerase (CRTISO) enzymes [11]. Z-ISO has been identified in arabidopsis and corn, and this enzyme catalyzes the isomerization of the PDS product 9,15,9′-tri-*cis*-*ζ* to 9,9′-di-*cis*-carotene [12,13]. Afterwards, ZDS converts 9,9′-di-*cis*-ζ-carotene to 7,9,7′,9′-tetra-*cis*-lycopene (prolycopene) through 7,9,9′-tri-*cis*-neurosporene [11]. CRTISO, a carotenoid isomerase, converts pro-lycopene to all-trans-lycopene. All-trans-lycopene produced through the CRTISO enzyme undergoes beta-lycopene cyclization to form all-trans-α-carotene and all-trans-*β*-carotene [14,15]. Through a series of reactions, all-trans-*α*-carotene is finally converted to lutein, and all-trans-*β*-carotene is converted to zeaxanthin and violaxanthin, during which, intermediates are converted to abscisic acid (ABA) and strigolactones (SL) by apocarotenoid enzymes [16]. CRTISO reported that the *ccr2* mutants in tomatoes and tangerines accumulate pro-lycopene and other poly-*cis*-carotene precursors in plastids of fruits [14,17]. In addition, *zebra2* mutants grown in a light-free environment accumulated pro-lycopene, and rice seedlings grown in a light-free environment showed reduced lutein levels and multi-colored phenotypes in leaves [18,19]. In addition, the *mit3* mutant showed phenotypes such as dwarfism, variegation in leaves, and an increase in the number of tillers [20]. It was considered that the increase in the number of tillers was caused by a decrease in the content of strigolactone which acts as the tiller inhibition function derived from the carotenoid pathway [20]. Recently, known gene editing technology using gene scissors has shown the potential to induce gene mutations at desired genomic DNA locations by using various types of site-specific nuclease. CRISPR/Cas9 (clustered regularly interposed short palindromic repeat (CRISPR)-associated protein (Cas) system), third-generation gene scissors, is a tool that can easily and accurately edit a target gene region [21]. Therefore, in this paper, we edited the *PHS3* (Os11g0572700) gene-encoding carotenoid isomerase of ABA biosynthesis using the CRISPR/Cas9 system. Our results suggested that the knockout lines of the *PHS3* gene would certainly help to not only reduction in ABA content but also PHS in rice, which will be helpful for elucidating the molecular mechanisms of PHS in other crops such as wheat and barley, which are susceptible to PHS.

## 2. Results

### 2.1. Confirmation and Expression of PHS3 Gene Encoding Carotenoid Isomerase in Rice

**Figure 1:**
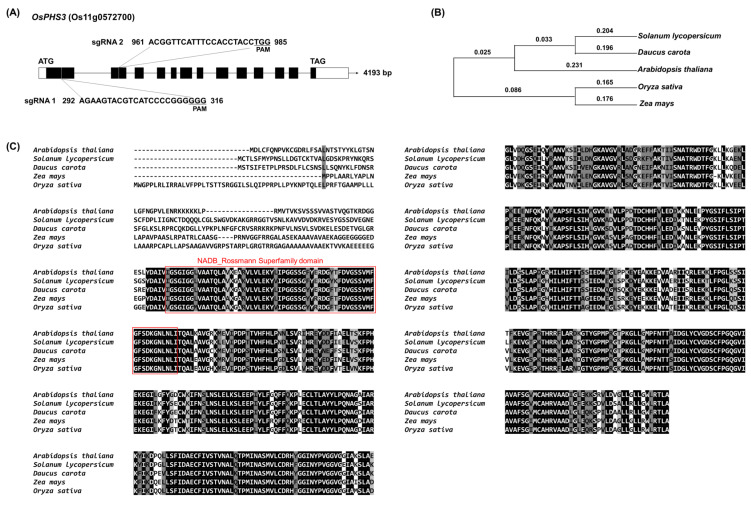
Genetic structure of *OPHS3* and similarity to other species. (**A**) Gene structure of *OsPHS3* gene (Os11g0572700) in rice. (**B**) Phylogenetic tree of *OsPHS3* gene sequences among species. (**C**) Multiple sequence alignments of PHS3 proteins identified with *Arabidopsis thaliana* (NM_100559), *Zea may* (NM_001367269), *Solanum lycopersicum* (NM_001309230), *Daucus carota* (NM_001329170). The red box indicates the NADB_Rossmann superfamily domain.

**Figure 2:**
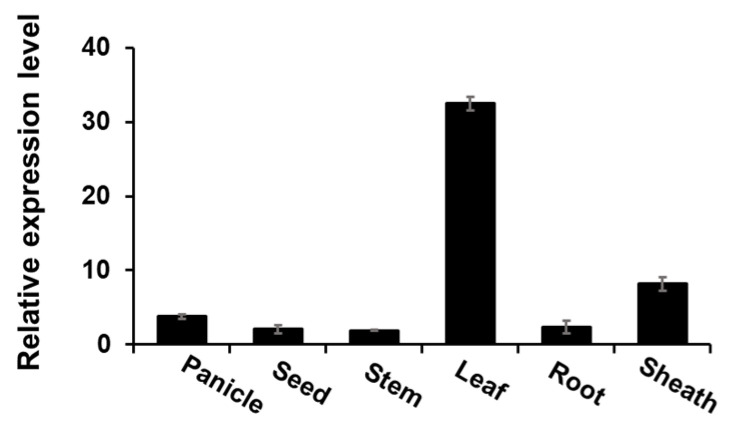
qRT-PCR analysis for gene expressions in various organs, including leaf, root, panicles, mature seed, and sheath. Error bars represent standard deviations calculated in three replicates (mean ± SD, *n*  = 3).

In rice, the *PHS3* gene is located on chromosome 11, the genomic sequence length is 4193 bp, consisting of 13 exons and 12 introns, and the CDS length is 1925 bp, encoding 641 amino acids (Figure 1). Also, genetic similarity between the *PHS3* gene sequences of various plant species showed high homology to *OsPHS3* and *Zea mays* (NM_001367269) and showed 87.83% identity at the amino acid sequence level. In addition, *Arabidopsis thaliana* (NM_100559), *Solanum lycopersicum* (NM_001309230), and *Daucus carota* (NM_001329170) showed 74.55–74.13% identity (Figure 1B). The conserved domain of the OsPHS3 protein has the NADB_Rossmann Superfamily containing amino oxidase, carotenoid isomerase, numerous dehydrogenases in metabolic pathways such as glycolysis, and GXGXGG patterns found in many oxidoreductases (Figure 1C). The expression level of the *OsPHS3* gene was analyzed in each organ using qRT PCR. As a result, the *OsPHS3* gene was expressed at a very high level in the leaves (Figure 2).

### 2.2. Generation of OsPHS3 Knockout Homozygous Mutants

**Figure 3:**
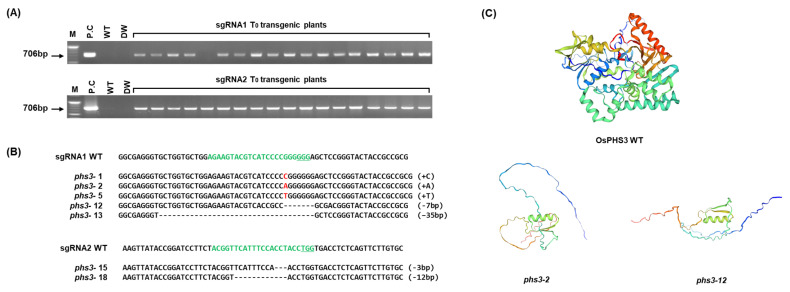
Production of *OsPHS3* transgenic plants using CRISPR/Cas9 system. (**A**) Selection T_0_ transgenic plants using bar-specific primers. M, 100bp marker; P.C, positive control. (**B**) Deep-sequencing analysis of the sgRNA target region. Green letters indicate sgRNA sequences. Deletion and insertion types were marked with dash and red letters, respectively. (**C**) Protein structure of OsPHS3 WT and mutant lines. The green helical cylinder structure seen in the mutant lines is the NADB_Rossmann Superfamily domain.

To investigate the in vivo function and role of the *OsPHS3* encoding carotenoid isomerase in rice, thirty-four transgenic plants were obtained by editing the first and second exons of *OsPHS3* in Dongjin as a target using the CRISPR/Cas9 system (Figure 1A and Figure 3A, Supplementary Figure S1). As a result of confirming the variation type of the target site through deep sequencing in the transformation-confirmed individuals, 18 out of 34 plants had targeted mutations, showing 53% gene editing efficiency, and the ratio of homozygous allelic 55.6%, heterozygous allelic 38.9%, and bi-allelic 5.6% (Supplementary Table S1). Of these, the selected *phs3-2* and *phs3-12* mutations were a 1 bp (A) insertion and a 7 bp deletion (GGGGGGA), respectively (Figure 3B). The DNA sequences of *OsPHS3* WT and two mutant lines were translated into amino acid sequences, and the protein structures were predicted. The results showed that 1 bp insertion and 7 bp deletion in the *OsPHS3* gene caused an early stop codon, which made complete protein production impossible. However, the NADB_Rossmann Superfamily domain was well conserved in amino acid sequence and protein structure (Figure 3C, Supplemental Figure S2). Therefore, the *phs3-2* and *phs3-12* mutants harvested T_1_ seeds by self-fertilization. In addition, we harvested T_2_ seeds from plants without amplified bands by PCR analysis using forward primers and reverse primers present in the *bar* gene region to select plants with T-DNA removed (Supplemental Figure S3). Harvested seeds were named *phs3-2* and *phs3-12* null lines, respectively, and were used in the following analyses.

### 2.3. Agronomic Traits of phs3-2 and phs3-12 Null Lines

**Figure 4:**
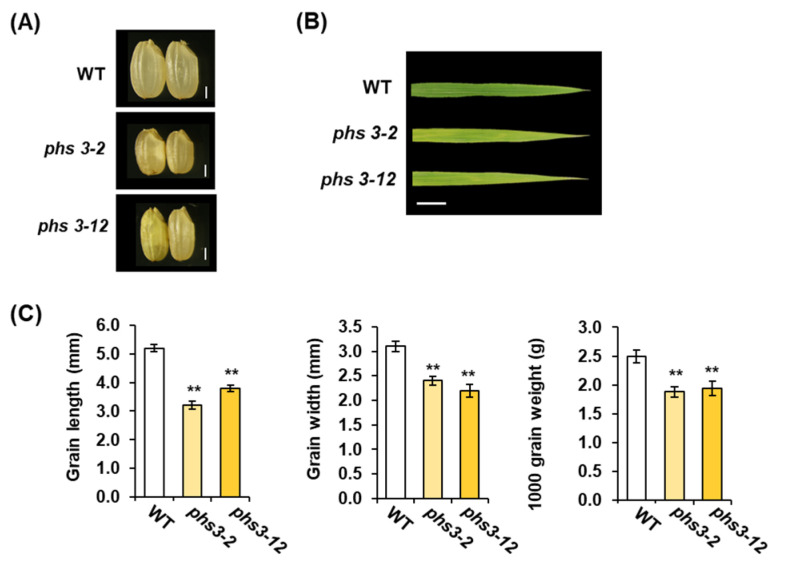
Phenotypes and agronomic traits of WT and *osphs3* mutant lines. (**A**) Morphology of mature seed. Bar = 1 mm. (**B**) Pale yellow-green variegated leaves of the *osphs3* mutant lines compared to WT. Bar = 1.5 cm. (**C**) Grain length, grain width, 1000 grain weight. Error bars represent standard deviations calculated in three replicates (mean ± SD, *n*  = 10). Asterisks show significant differences (** *p*  <  0.05) between *osphs3* null lines and WT.

To observe the growth of *phs3-2* and *phs3-12* null lines in a rice field, major agricultural traits such as plant height, culm diameter, panicle length, and number of tillers were investigated. The *phs3*-2 and phs3-12 null lines showed decreased plant height, stem diameter, and spike length compared to WT, but increased tiller numbers compared to WT (Table 1). In addition, the seed size and seed weight of the phs3-2 and phs3-12 null lines decreased compared to WT (Figure 4C). The *phs3-2* and *phs3-12* null lines showed a distinctly pale yellow-green phenotype in the leaf (Figure 4B).

### 2.4. Carotenoid Profiles of phs3-2 and phs3-12 Null Lines

**Figure 5:**
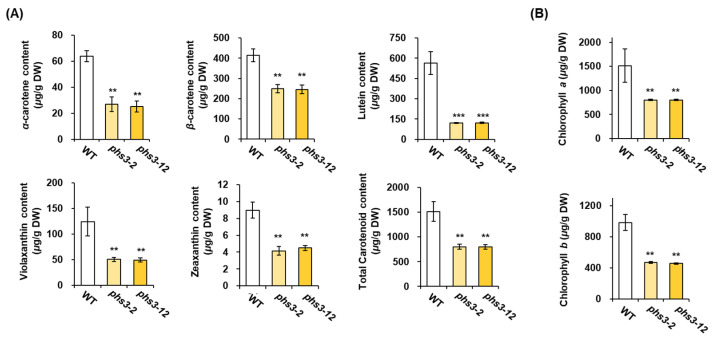
Quantification of carotenoid and chlorophyll levels in leaves of WT and *osphs3* null lines using HPLC analysis. Error bars represent standard deviations calculated in three replicates (mean ± SD, *n*  = 3). Asterisks show significant differences (** *p*  <  0.05, *** *p* < 0.01) between *osphs3* null lines and WT.

To analyze the carotenoid metabolisms of *phs3-2* and *phs3-12* null lines, HPLC analysis was performed with extracts from null lines and WT grown under light conditions. Phytoene, zeta-carotene, and pro-lycopene were accumulated in the leaves of the *phs3-2* and *phs3-12* null lines (Figure 5). Also, the *α*-carotene, *β*-carotene, lutein, violaxanthin, and zeaxanthin, which are carotenoid substances after carotenoid isomerase converts pro-lycopene into all-trans lycopene to form a cyclization substrate, were measured and compared (Figure 5). In all the items examined, *phs3-2* and *phs3-12* null lines were reduced by 2 to 4 times than those of WT.

### 2.5. Expression Profiling of Carotenoid Biosynthesis Genes

**Figure 6:**
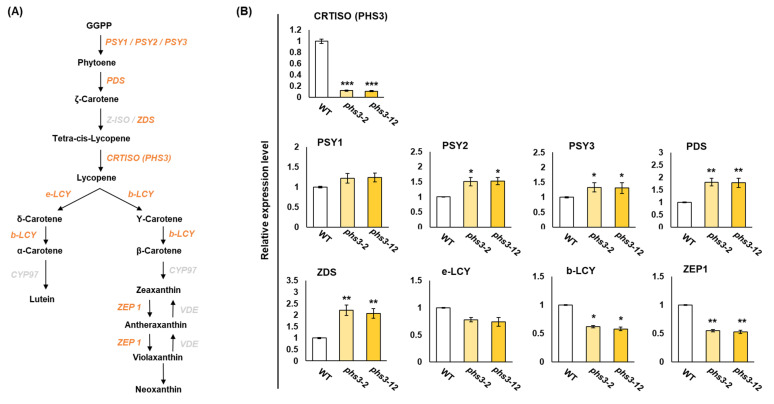
Quantification of relative expression levels of genes involved in carotenoid biosynthesis pathway in *osphs3* null lines and WT using qRT-PCR analysis. (**A**) Carotenoid biosynthetic pathway in rice. Orange and gray letters represent genes involved in producing carotenoid products (black letters), and genes analyzed in this experiment are shown in orange letters. (**B**) Results of qRT-PCR analysis for relative gene expression levels. Error bars represent standard deviations calculated in three replicates (mean ± SD, *n*  = 3). Asterisks show significant differences (* *p*  <  0.1, ** *p*  <  0.05, *** *p* < 0.01) between *osphs3* null lines and WT.

To investigate the expression of genes placed on the circuit due to *OsPHS3* gene knockout in the carotenoid biosynthesis pathway, we performed qRT-PCR analysis on a total of nine genes including *PSY1*, *PSY2*, *PSY3*, *PDS*, *ZDS*, *CRTISO*, ε*-LCY*, *β* -*LCY* and *ZEP1*. As a result, gene expressions involved in *PSY1*, *PSY2*, *PSY3*, *PDS,* and *ZDS* in the *phs3-2* and *phs3-12* null lines tended to be higher than those in WT. However, in the rest of the genes, the *phs3-2* and *phs3-12* null lines showed lower expression levels compared to WT (Figure 6).

### 2.6. ABA and Pre-Harvest Sprouting in phs3-2 and phs3-12 Null Lines

**Figure 7:**
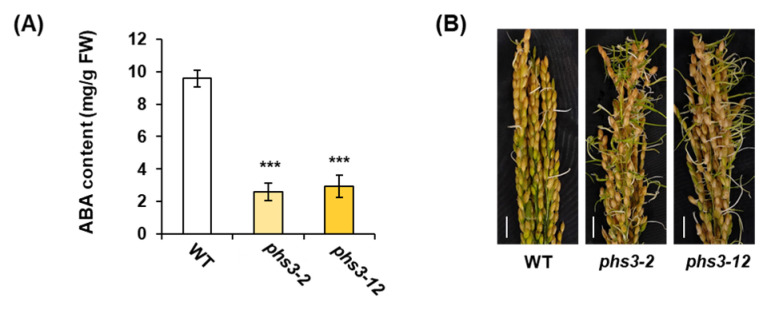
ABA Contents and pre-harvest sprouting rate of WT and *osphs3* null lines. (**A**) Analysis of ABA content in WT and *osphs3* null line seeds. Error bars represent standard deviations calculated in three replicates (mean ± SD, *n*  = 3). Asterisks show significant differences (*** *p* < 0.01) between *osphs3* null lines and WT. (**B**) Pre-harvest sprouting rate of WT and *osphs3* null lines. Bar = 3 cm.

To investigate whether the reduced level of carotenoids in *phs3-2* and *phs3-12* null lines causes a decreased level of ABA, we measured the ABA content by immunoassay. The amount of ABA in *phs3-2* and *phs3-12* null lines was reduced compared to the wild type. In addition, in pre-harvest sprouting experiments, the germination rates of *phs3-2* and *phs3-12* null lines were significantly increased compared to WT (Figure 7).

## 3. Discussion

PHS is a phenomenon in which rice, wheat, and barley sprout from ears before harvesting due to frequent rainfall and loading in the early ripening season [3,22]. These phenomena refer to the breakdown and dissolution of germination inhibitory substances in ears exposed to excessive humidity conditions, breaking the seed’s dormancy and progressing germination [23]. Additionally, factors closely related to PHS include external factors such as environmental stress, cultivation conditions, harvest time, day length, and temperature during ripening, as well as internal factors such as seed dormancy, seed permeability, amylase activity, and endogenous hormone level [24,25]. Among them, seed dormancy occurs through interactions with plant hormones such as ABA and external factors such as field conditions or temperature during the maturation of seeds [26]. Thus, embryonic ABA not only plays a central role in inducing and maintaining seed dormancy, but also has mechanisms that inhibit the transition from embryo to germination. The CRTISO protein in the carotenoid biosynthesis pathway isomerizes pro-lycopene to convert it into all-trans lycopene, through which precursors such as *α*-carotene, *β* carotene, lutein, and zeaxanthin are synthesized [15]. In several plants, such as *Arabidopsis thaliana*, *Zea mays*, *Solanum lycopersicum*, and *Daucus carota*, CRTISO functions as carotenoid isomerase, amino oxidase, and has NADB_Rossmann Superfamily domains during carotenoid biosynthesis [15]. In this study, we used the CRISPR/Cas9 system to knockout of the *PHS3* gene encoding carotenoid isomerase in Dongjin rice and showed non-lethal and ’variegated’ phenotypes (Figure 4 and Figure 7). In the leaves grown on *phs3-2* and *phs3-12* null lines, *α*-carotene, *β* carotene, lutein, and zeaxanthin were dramatically reduced, and pro-lycopene accumulated (Figure 5). These results were previously consistent with those of *crtiso* variants of tomato and *Arabidopsis*. Interestingly, CRTISO activity could explain the survival of *phs3* mutants by photoisomerization [27,28]. Usually, membranes in the form of chlorophyll–carotenoid–protein complexes and some carotenoid enzymes are associated with membranes, and changes in carotenoid composition or carotenoid enzymes themselves always lead to abnormal pigmentation [8,14,29]. In addition, CRTISO enzyme activity was lost in the *ccr2 Arabidopsis* mutant, showing a variegated phenotype [14]. Furthermore, qRT-PCR analysis of nine genes presents in the carotenoid biosynthetic pathway in the present study using *phs3-2* and *phs3-12* null lines showed that the expression level of genes producing PSY1, PSY2, PSY3, PDS, and ZDS was high, but the expression of all genes belonging to the next stage decreased. These results were identical to the changes in carotenoid metabolites (Figure 6). In addition, in this study, we investigated how the reduction in carotenoids affects ABA content and pre-harvest sprouting. As a result, the ABA content in *phs3-2* and *phs3-12* null lines was lower than that of WT, and it was sensitive to pre-harvest sprouting (Figure 7). Previous studies have suggested that ABA-insensitive arabidopsis mutants have reduced seed maturity and dormant status. However, ABA-deficient mutations in corn suggest that ABA absolute levels do not always correlate perfectly with dormancy and germination, but that other regulatory factors are involved together [30]. Therefore, viviparous mutants in rice are ideal for elucidating the complex mechanisms of pre-harvest sprouting and dormancy. Therefore, a detailed comparison of the differential expression patterns between the different kinds of *osphs3* mutants and WT involved in the carotenoid biosynthesis pathway is thought to be able to identify the major factors regulating PHS. Therefore, the KO lines obtained in this study are thought to be able to assess whether ABA metabolism is highly regulated at the temporal and spatial levels during seed development. In addition, we look forward to more information about the physiological function of carotenoids and the molecular mechanisms of PHS in the future.

## 4. Materials and Methods

### 4.1. Plant Materials and Growing Conditions

Dongjin (*Oryza sativa* L., ssp. Japonica) used in the study was used for breeding transgenic plants. Transgenic plants were grown in GMO-free greenhouses after acclimation [31]. After harvesting the seeds, the moisture was dried to 14% and then stored at a temperature of 4 °C.

### 4.2. Selection of Gene Target Site and CRISPR/Cas9 Vector Construction

The *PHS3* gene (accession no. BAF28490), which synthesizes all-trans-lycopene in the rice carotenoid biosynthesis pathway, has genomic information (Gene: Os11g0572700) from Gramene site (https://www.gramene.org/, accessed on 6 January 2020 [32]. The sgRNAs were selected using CRISPR RGEN Tools (http://www.rgenome.net/cas-designer/, accessed on 23 January 2020) with the *PHS3* gene information [32,33]. The CRISPR/Cas9 vectors were constructed to express sgRNA such as OsU3:: *OsPHS3*-sgRNA/pBOsC according to the previously reported method [33], and the flanking sequence was confirmed by the Sanger sequencing method [34].

### 4.3. Generation and Selection of Transgenic Rice Plants

Rice transformation was performed according to the method of Nishimura et al. (2006) [35]. Rice seed-derived callus tissues were infected using *Agrobacterium tumefaciens* strain EHA105. Prior to the transplantation of the transgenic plants into the soil, a selection medium containing 6 mg/liter phosphinotrisin and 400 mg/liter carbenicillin was used. Total DNA extraction was performed by sampling 100 mg of leaves and using a DNA Quick Plant Kit (Inclone, Jeonju, Republic of Korea). Selection of T-DNA-inserted transgenic plants was performed using bar gene-specific primers (Bar-F 5′−CGTCAACCACTACATCGAGA−3′) and (Bar-R 5′−AAGTCCAGCTGCCAGAAA−3′), under PCR conditions in which pre-denaturation was performed at 95 °C for 5 min, denaturation was performed at 95 °C for 30 s, extension was repeated at 56 °C for 30 s, and extension was performed at 72 °C for 7 min. Selection of T-DNA-inserted transgenic plants was carried out using bar gene-specific primers (Bar-F 5′−CGTCAACCACTACATCGAGA−3′) and (Bar-R 5′−AAGTCCAGCTGCCAGAAA−3′), and PCR conditions were carried out according to previously reported methods [35].

### 4.4. Deep Sequencing Analysis of Target Genes

PCR analysis was performed using a PCR master mix (Bioneer, Daejeon, Republic of Korea) with genomic DNA (50 ng/μL) as a template to identify mutations at the target site. PCR amplicons were performed using MiniSeq (Illumina, San Diego, CA, USA) paired-end read sequencing, and next-generation sequencing (NGS) data derived from MiniSeq were performed using Cas-Analyzer (https://www.rgenome.net/cas-analyzer, accessed on 16 July 2024), which was previously reported by Park et al., 2017 [36].

### 4.5. Carotenoid Extraction and HPLC Analysis

Carotenoid extraction from WT and transgenic rice leaves was performed by extracting 100 mg lyophilized with acetone (0.01% BHT). The extract was centrifuged at 5640× *g* (Eppendorf, 5430R, Hamburg, Germany) for 30 min at 4 °C conditions and filtered through a 0.45 μm PTFE syringe filter (Whatman, New York, NY, USA). An Agilent 1260 high-performance liquid chromatography (HPLC) system (Hewlett-Packard, Waldbronn, Germany) was used according to the previously reported method [37]. Thereafter, Chemstation software (Agilent, Santa Clara, CA, USA) was used for the collection and analysis of HPLC. ESI-MS spectra were obtained using an API 4000TM Triple Quadrupole Mass Spectrometer (SCIEX, Concord, ON, Canada). The source temperature was 300 °C, and the ion spray voltage was performed at 5500 V. Gas 1 and 2 settings for nitrogen (>99.999%) were set to 60 and 70, respectively.

### 4.6. Gene Expression Profiling by qRT-PCR Analysis

Total RNA was extracted by sampling 100 mg of 2-week-old plant leaves using a plant total RNA mini-kit (Cat. FAPRK 001-1). Extracted total RNA was synthesized with cDNA using a reverse transcription system (Takara, www.takara-bio.com). qRT-PCR analysis was performed under conditions previously reported by a Light Cycler 480 device (Roche, Pleasanton, CA, USA) with a total volume of 20 μL using cDNA, primers, and SYBR Green Real-time PCR Master Mix (Toyobo, https://www.bio-toyobo.cn) at a concentration of 50 ng/μL [31]. The housekeeping gene *OsActin* (Os11g0163100) was used to quantify expression levels. Relative quantification of transcript levels was performed using the comparative Ct method [38].

### 4.7. Test of Pre-Harvest Sprouting

After pollinating the *phs3-2* and *phs3-12* null lines for pre-harvest sprouting tests, the panicles of 35-day-old plants were collected and immersed in water for 4 h. After that, the cotton swab was sufficiently immersed in water, placed on a tray, and the dipped rice panicles were placed on the cotton swab and incubated at 28 °C for 24 h under light conditions. The wet swab was replaced every 24 h to minimize contamination. The seed germination rates of the panicles were examined on days 4, 6, 8, and 11, respectively.

### 4.8. Determination of ABA

Determination of ABA with 2-week-old seedlings was performed as previously described [39].

### 4.9. Statistical Analysis

The data collected in this study were analyzed by one-way analysis of variance (ANOVA) using the statistical analysis system (SAS version 9.4). Data values were mean ± SE (*n* = 3) and statistical significance was set to *p* < 0.05, and post-analysis was performed according to Duncan’s multiple range test [40].

## Figures and Tables

**Table 1:** Agronomic traits of *phs3-2* and *phs3-12* null lines.

Line	Plant Height (cm)	Culm Diameter (mm)	Panicle Length (cm)	Number of Tillers
WT	119.8 ± 10.4	6.1 ± 0.13	22.58 ± 0.86	13.8 ± 0.08
*phs3-2*	82.3 ± 6.7	4.7 ± 0.12	20.41 ± 0.47	16.2 ± 0.04
*phs3-12*	84.1 ± 3.4	4.6 ± 0.20	21.09 ± 0.55	16.3 ± 0.02

The values represent mean ± SD (*n* = 3).

## Data Availability

The original contributions presented in the study are included in the article/Supplementary Materials, further inquiries can be directed to the corresponding authors.

## Supplementary Materials

The following supporting information can be downloaded at: https://www.mdpi.com/article/10.3390/plants14030345/s1, Figure S1: Schematic representation of CRISPR/Cas9-mediated targeted mutagenesis of *OsPHS3*. (A) T-DNA region of the recombinant OsU3:: *OsPHS3*-sgRNA/pBOsC vector carrying *OsPHS3*-sgRNA under the control of the OsU3 promoter. (B) *Agrobacterium*-mediated plant transformation. a, seed sowing; b, callus induction; c, infection; d, infected callus proliferation; e, shoot induction; f, shoot differentiation; g, root induction; h, transplantation into pots.; Figure S2: PCR analysis using bar-specific primers for selection of null gene-edited plants; Table S1: *OsPHS3* transgenic plant production rate and editing type and ratio; Table S2: The primers list used in this study.
