# Supplementary material for: ABA and Pre-Harvest Sprouting Differences in Knockout Lines of OsPHS3 Encoding Carotenoid Isomerase via CRISPR/Cas9 in Rice
Source: Plants (Basel). 2025 Jan 23;14(3):345. doi: 10.3390/plants14030345 (PMC11820545; doi:10.3390/plants14030345)
Supplement: Supplementary file 1 [file plants-14-00345-s001.zip › plants-3393373-supplementary.pdf]

Supplementary Data

ABA and Pre-Harvest Sprouting Differences in Knockout Lines of OsPHS3 Encoding Carotenoid Isomerase via CRISPR/Cas9 in Rice

Ji-Yun Go [1†], Jin Young Kim [2†], Hyo Ju Lee [2], Jong Hee Kim [2], Hye Mi Lee [2], Yong Gu Cho [3], Kwon Kyoo Kang [2,4,*] and Yu Jin Jung [2,4,*]

Contents

Table S1. *OsPHS3* transgenic plant production rate and editing type and ratio.

Table S2. The primers list used in this study.

Figure S1. Schematic representation of CRISPR/Cas9-mediated targeted mutagenesis of *OsPHS3*. (A) T-DNA region of the recombinant OsU3:: *OsPHS3*-sgRNA/pBOsC vector carrying *OsPHS3*-sgRNA under the control of the OsU3 promoter. (B) *Agrobacteirum*-mediated plant transformation. a, seed sowing; b, callus induction; c, infection; d, infected callus proliferation; e, shoot induction; f, shoot differentiation; g, root induction; h, transplantation into pots.

Figure S2. Amino acid sequences of OsPHS3 WT and mutant lines. Underline indicates amino acid sequences that have changed compared to WT, and asterisk indicates a stop codon.

Figure S3. PCR analysis using bar-specific primers for selection of null gene-edited plants.

Supplementary Table S1. *OsPHS3* transgenic plant production rate and editing type and ratio.

Gene Name	No. of T_0 plant	Edited plants (%)	Deep seq analysis (NGS)		
			Homozygous (%)	Bi-allelic (%)	Heterozygous (%)
OsPHS3	34	18(53.0%)	10/18(55.6%)	1/18(5.6%)	7/18(38.9%)

Supplementary Table S2. The primers list used in this study.

Primer name	Sequence (primer direction 5′-3′)	purpose
J67 pBOsC sgSEQ - FW	CAGCTTGGCTCTAGTCGACC	
K20 RGEN scaaffold regoin RV	CGGTGCCACTTTTTCAAGTT	
OsPHS3 sg1 up	ggcagAGAAGTACGTCATCCCCGGG	Vector construction
OsPHS3 sg1 down	aaacCCCGGGGATGACGTACTTCTc	
OsPHS3 sg2 up	ggcagACGGTTCATTTCCACCTACCTGG	
OsPHS3 sg2 down	aaacCCAGGTAGGTGGAAATGAACCGTc	
T-DNA confirm-Nos ter Fw	TTGCGCGCTATATTTTGTTTT	T-DNA confirm
T-DNA confirm-Bar R Rv	CGTCAACCACTACATCGAGA	
OsPHS3 sg1 1st F1	CGCCGGAGCAGTAGTAGG	
OsPHS3 sg1 1st R1	CTTTCCCTTCCCCAATTCTT	
OsPHS3 sg1 2nd F1	ACACTCTTTCCCTACACGACGCTCTTCCGATCTGGAGGGTGGGGGAGAGTA	
OsPHS3 sg1 2nd R1	GTGACTGGAGTTCAGACGTGTGCTCTTCCGATCTATCTACACCCAACCGTGCTC	deep-sequencing
OsPHS3 sg2 1st F1	ATAAAAACCCCACCCAGCTC	
OsPHS3 sg2 1st R1	CGTCTAGCCAACAACATCCA	
OsPHS3 sg2 2nd F1	ACACTCTTTCCCTACACGACGCTCTTCCGATCTAGGAGGGTGGGGGAGAGTA	
OsPHS3 sg2 2nd R1	GTGACTGGAGTTCAGACGTGTGCTCTTCCGATCTTCACTGTCCTCACCTTGTCG	
OsACTIN FW	CAACACCCCTGCTATGTACG	
OsACTIN RV	ATCACCAGAGTCCAACACAA	
OsPHS3 qRT-PCR Fw	TGCCTGCTGATACTGATTGC	qRT-PCR analysis
OsPHS3 qRT-PCR Rv	ATGGGTCAAGCACTGTAGGG	
OsPSY1 qRT-PCR Fw	GTCTGGGCGTCTCTGTTGTT	
OsPSY1 qRT-PCR Rv	CGCCCTCTTTGTGAAGTTGT	

OsPSY2 qRT-PCR Fw	TGATGCTATCGAAGCAAACG
OsPSY2 qRT-PCR Rv	GCATAAGCGACCGGTAAAGA
OsPSY3 qRT-PCR Fw	GAGATCGAGGCCAACGATTA
OsPSY3 qRT-PCR Rv	GGGGAGCATGAGTGATCTGT
OsPDS qRT-PCR Fw	GGTTGCAATGGAAGGAACAC
OsPDS qRT-PCR Rv	CATTTAAGGGTGCAGGCAAT
OsZDS qRT-PCR Fw	GCGAGTCACCAGGAAATGAT
OsZDS qRT-PCR Rv	CCCTTCCATGCTGTCAATGT
OsLCY B qRT-PCR Fw	TGGACATCCTCCTCAAGCTC
OsLCY B qRT-PCR Rv	AAGAAGAGCCTCGACGACAG
OsLCY E qRT-PCR Fw	CTTGGTTCGACGCTTTCTTC
OsLCY E qRT-PCR Rv	GGTTCATTCGCATTTGGTTC
OsZEP1 qRT-PCR Fw	ACCGACAACGAAGGTAGACG
OsZEP1 qRT-PCR Rv	CGGCCTTTTTATCTGAACCA

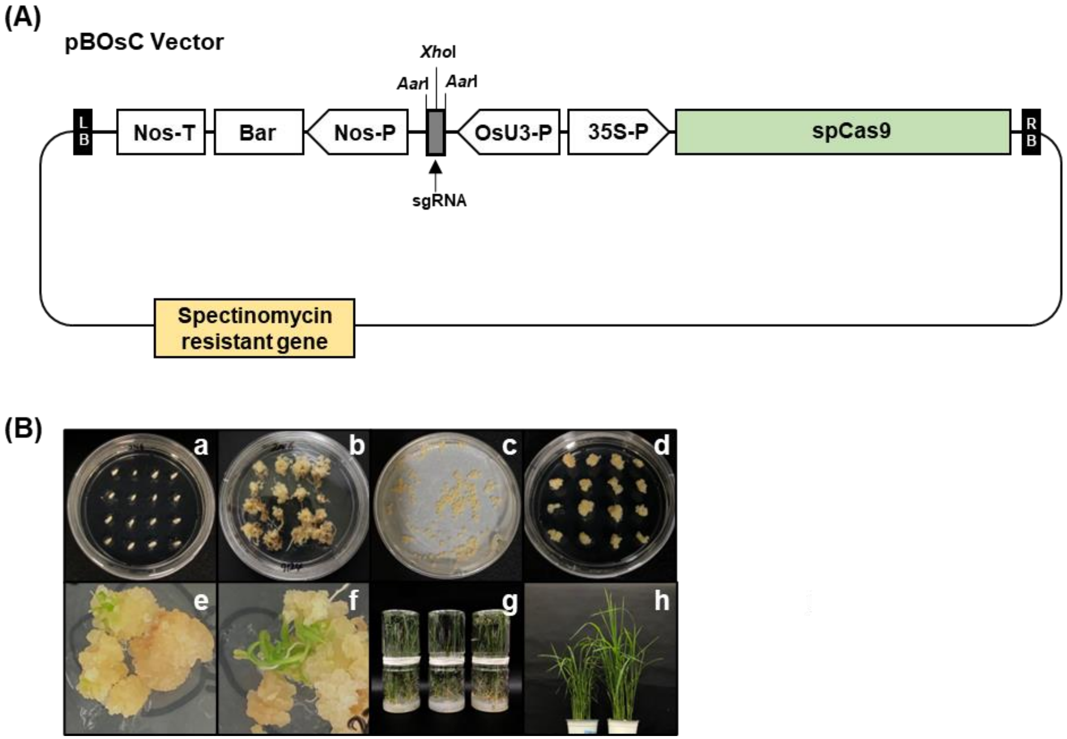

Supplementary Figure S1. Schematic representation of CRISPR/Cas9-mediated targeted mutagenesis of *OsPHS3*. (A) T-DNA region of the recombinant OsU3::*OsPHS3*-sgRNA/pBOsC vector carrying *OsPHS3*-sgRNA under the control of the OsU3 promoter. (B) *Agrobacteirum*-mediated plant transformation. a, seed sowing; b, callus induction; c, infection; d, infected callus proliferation; e, shoot induction; f, shoot differentiation; g, root induction; h, transplantation into pots.

WT amino acid sequence

MPLLLLAAARPCAPLLAPSAAGAVVGRPSTARPLGRGTRRGAGAAAAAAVAAEKTVVKAEEEEEEGGGEYDAIVVGSGIGGMVAATQLAAKGARVLVLEKYVIPGGSS
GYYRRDGFTFDVGSSVMFGFSDKGNLNLITQALEAVGHKMEVIPDPSTVHFHLPGDLSVLVHREYDDFVTELVNKFPHEKEGILKFYGTCWKIFNSLNSLELKSLEE
PLYLFGQFFQKPLECLTLAYYLPQNAGDIARKFIKDQQLLSFIDAECFIVSTVNALQTPMINASMVLCDRHFGGINYPVGGVGGIAMSLADGLVDKGSEIRYKANVT
NVILENGKAVGVRLSNGKEFFAKTVISNATRWDTFGKLLKVEELPEEEKNFQKNYVKAPSFLSIHMGVKASVLPADTDCHHFVLEDDWANLEKPYGSIFLSIPTVLD
PSLAPEGHHILHIFTTAGIEDWEGLSRKDYEKKKELVATEIIKRLEKKLFPGLQDSIVLKEVGLTKNTPKISCAK*

***phs3-2* amino acid sequence**

MPLLLLAAARPCAPLLAPSAAGAVVGRPSTARPLGRGTRRGAGAAAAAAVAAEKTVVKAEEEEEEGGGEYDAIVVGSGIGGMVAATQLAAKGARVLVLEKYVIPRGEL
RVLPPRRVHLRRRLLRHVRLLRQGELEFDYTSTRSSWA*

***phs3-12* amino acid sequence**

MPLLLLAAARPCAPLLAPSAAGAVVGRPSTARPLGRGTRRGAGAAAAAAVAAEKTVVKAEEEEEEGGGEYDAIVVGSGIGGMVAATQLAAKGARVLVLEKYVIPAPGT
TAATGSPSTSAPPSCSASPTRGT*

Supplementary Figure S2. Amino acid sequences of OsPHS3 WT and mutant lines. Underline indicates amino acid sequences that have changed compared to WT, and asterisk indicates a stop codon.

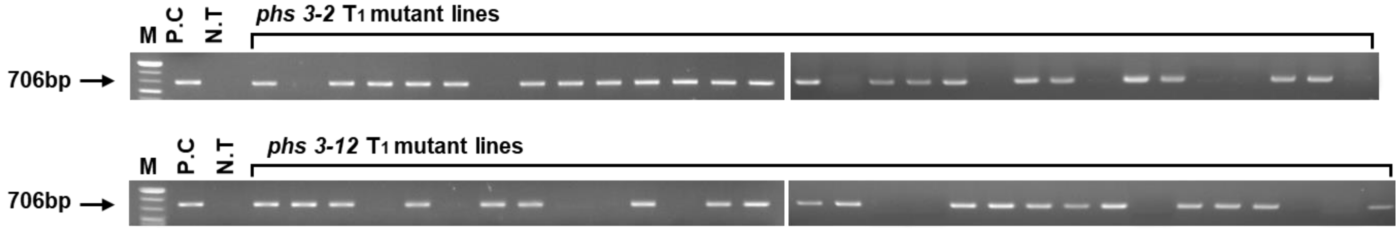

Supplementary Figure S3. PCR analysis using bar-specific primers for selection of null gene-edited plants.
